# Dialects in leaf-clipping and other leaf-modifying gestures between neighbouring communities of East African chimpanzees

**DOI:** 10.1038/s41598-022-25814-x

**Published:** 2023-01-05

**Authors:** Gal Badihi, Kirsty E. Graham, Brittany Fallon, Alexandra Safryghin, Adrian Soldati, Klaus Zuberbühler, Catherine Hobaiter

**Affiliations:** 1 School of Psychology and Neuroscience, University of St Andrews, St Andrews, UK; 2 Department of Comparative Cognition, Institute of Biology, University of Neuchâtel, Neuchâtel, Switzerland; 3 Budongo Conservation Field Station, Masindi, Uganda

**Keywords:** Cultural evolution, Animal behaviour

## Abstract

Dialects are a cultural property of animal communication previously described in the signals of several animal species. While dialects have predominantly been described in vocal signals, chimpanzee leaf-clipping and other ‘leaf-modifying’ gestures, used across chimpanzee and bonobo communities, have been suggested as a candidate for cultural variation in gestural communication. Here we combine direct observation with archaeological techniques to compare the form and use of leaf-modifying gestures in two neighbouring communities of East African chimpanzees. We found that while both communities used multiple forms, primarily within sexual solicitation, they showed a strong preference for a single, different gesture form. The observed variation in form preference between these neighbouring communities within the same context suggests that these differences are, at least in part, socially derived. Our results highlight an unexplored source of variation and flexibility in gestural communication, opening the door for future research to explore socially derived dialects in non-vocal communication.

## Introduction

Evidence for the presence of culture in non-human animals is increasingly rich and widespread^1,2^, particularly in non-human primates^2–4^. In non-human animals, culture is typically defined as the variation in behaviour between groups or populations arising through social learning processes and persisting over generations^2,5,6^. Cultural variation has been identified across several behavioural domains—most prominently tool-use^3^ and food processing^7^; but also including social customs^6^, and, more recently, communication^8,9^. The method of exclusion^2^ is a common methodology used to identify a cultural trait. Here, by ruling out the possibility that variation between groups in a specific trait is due to ecological or genetic differences, social learning is assumed to be the most likely explanation of variation, suggesting that the trait is cultural^2^. For many behaviour, this method has been applied by showing variation between neighbouring or near-by communities of chimpanzees who occupy territories with similar ecology and experience regular gene flow between them^6,7^.

The term ‘dialect’ has been broadly used to refer to variation in the communication between groups of both human and non-human animals^10,11^. In some species, dialects are socially derived, as individuals within the same social group conform to the same communicative variants e.g.,^1,12^; here, a dialect may also be considered a cultural trait. While the majority of studies exploring dialects in non-human animals have focused on vocal communication^8,10,12,13^, this field of study has also expanded to include human sign language^14^ and non-vocal signals such as the honeybee waggle dance^15^. Comparisons of vocal and/or auditory signals between groups typically involve the comparison of quantitative features of signals (e.g., fundamental frequency^12,16^), which can be compared systematically across studies using established methods. However, there are no similar systematic descriptions of how features in non-vocal signals might vary, or at what scale to compare them. As a result, comparison of non-vocal signals between communities is challenging; however, ape gestures—and more specifically leaf-clipping—have been suggested to be a promising area for potential inter-group variation.

Leaf-clipping is a non-vocal signal, used by chimpanzees and bonobos, that has been suggested as a potential cultural candidate, supported by the variation in its presence across communities^2,17^ (Table 1), and by possible variation in its expression as well as the behavioural contexts in which is it used^2,18–22^. It has also been suggested as an example of gestural dialects in chimpanzees^23^. While some researchers have described leaf-clipping as a type of tool use^18,19,22^, descriptions of a behaviour as tool-use or signal are not mutually exclusive^22^—an object used to produce a gesture may also be considered a communicative tool. Nonetheless, a systematic investigation of this behaviour between neighbouring communities living in the same forest environment and with regular gene flow has not yet been attempted (c.f. Bessa et al.^18^ for an initial description).

**Table 1:**
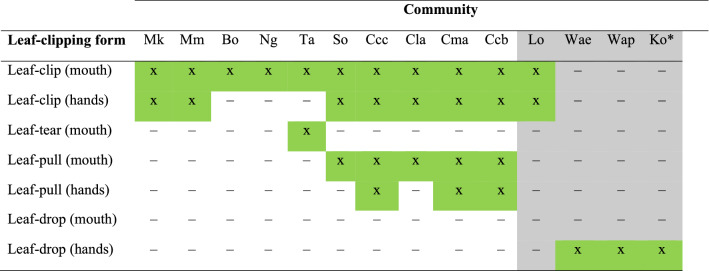
Leaf-modifying gesture forms described in different communities of chimpanzees and bonobos in the current literature.

“X” in a green cell indicates this behaviour is confirmed in the community “–” indicates behaviour is not confirmed. White columns and grey columns indicate chimpanzee and bonobo communities, respectively.

*Codes for each community:

*Chimpanzees.*

Mk = K group, Mahale, United Republic of Tanzania^2^.

Mm = M group, Mahale, United Republic of Tanzania^20^.

Bo = Bossou, Bossou, Republic of Guinea^21^.

Ng = Ngogo, Kibale, Republic of Uganda^22^.

Ta = South Group, Taï, Republic of Côte d'Ivoire^19,25^.

So = Sonso, Budongo, Republic of Uganda^29^.

Ccc = Caiquene-Cadique group, Cantanhez National Park, Republic of Guinea-Bissau^20^.

Cla = Lautchandé group, Cantanhez National Park, Republic of Guinea-Bissau^20^.

Cma = Madina group, Cantanhez National Park, Republic of Guinea-Bissau^20^.

Ccb = Cambeque group, Cantanhez National Park, Republic of Guinea-Bissau^20^.

*Bonobos.*

Lo = Eyengo community, Lomako, Democratic Republic of the Congo^17^.

Wae = E1 community, Wamba, Democratic Republic of the Congo^26^.

Wap = P community, Wamba, Democratic Republic of the Congo^28^.

Ko = Kokoalongo, Ekalakala, and Fekako communities, Kokolopori, Democratic Republic of the Congo –* data are for one population that included three communities^29^.

Leaf-clipping was first described as a gesture in the Mahale chimpanzee community in Tanzania^24^. The behaviour was defined as: “An individual holds one to five leaves by the petiole between their thumb and index finger, then pulls the leaves from side to side while ripping off the blade with the incisors”^24^. In this first published description, Nishida speculated that leaf-clipping may represent an example of ‘traditional drift’^24^. Further discussions and descriptions of leaf-clipping developed this idea and considered the trait to be likely cultural due to apparent variation in use and expression between communities^2,18,19^. Across communities, this behaviour primarily occurs in sexual contexts and is argued to function as a courtship behaviour to solicit copulations, particularly by young males toward females in oestrus^19,22,24^; however, it is also observed preceding buttress drumming^18,19,22,25^. Since this initial definition, other variants of this behaviour—also labelled as “leaf-clipping” —have been described such as the tearing of leaves using both hands instead of hand and mouth^2,18^ and the tearing of leaves at different locations on the leaf^19^. In addition, leaf modification was also observed in apparently similar gestures in the same behavioural contexts as leaf-clipping, but these variants were given new names by the researchers who first described them. In Cantanhez National Park chimpanzees use ‘leaf-pull’ gestures as well as leaf-clipping gestures before buttress drumming^18^,—although, as data at this site were recorded using camera traps, these gestures may also occur in other contexts that were not captured on video. Bonobos (*Pan paniscus*) at Wamba and Kokolopori have been described to use ‘leaf-drop’ or ‘drop twig’ gestures to solicit others for sex^26,27^.

While previous reports and discussions of leaf-clipping highlight apparently cultural differences between communities^2,19,24^, these descriptions did not control for genetic or ecological variation between communities that could explain differences in behaviour type or expression (but see Bessa et al.^18^). Establishing whether there are consistent patterns of variation that can be distinguished between individuals, groups, or contexts relies on being able to reliably discriminate distinct forms; however, brief descriptions and a lack of video examples of leaf-clipping and other leaf-using gestures have made it difficult to determine whether the different observations describe the same or several behaviours. Furthermore, these observations can be relatively rare and may be absent for years at a time in some groups^25^. Previous reports of these gestures either included them in addition to other behaviour^19,21,22^, or provide detailed descriptions of specific observations but without analysing the function or variation of these gestures^20,24^.

Given the similarity across descriptions of leaf-clipping and the overlap in behavioural contexts in which leaf-clipping and other leaf-modifying gestures are observed, we propose that an appropriate null hypothesis is that these gesture forms represent variants within a ‘leaf-modifying gesture’ category. In doing so, we do not make the a priori assumption that the variation in the way in which these gestures are produced (their form) necessarily means they represent distinct gesture ‘types’ or categories from the perspective of the chimpanzees using them^28^. We follow the widely used definition of ‘gestures’ as mechanically ineffective actions of the limbs or body produced in a goal-directed, intentional way^26,28–32^. Throughout this paper, we use the term ‘leaf-modifying gestures’ for the category of gestures in which individuals modify leaves and small leafy stems as an inherent part of the gesture action, for example through the tearing/ripping or removal of leaves or small leafy stems. We did not consider other object-related gesture actions that may incidentally modify objects, including leaves, but that do not aim to modify these objects as part of their action (e.g., ‘Object shake’, ‘Object Hit’, or ‘Throw’ may on occasion modify the object they are manipulating but it is not a requirement of their production)^29^. The leaf-modifying gestures described in this study are also distinct from other leaf-modifying behaviour (e.g., leaf-groom, leaf-inspect) not used during gestural communication.

Four forms of leaf-modifying gestures have been reported in great ape communication: leaf-clip, leaf-tear, leaf-pull, leaf-drop. While in some cases multiple forms have been labelled under the term ‘leaf-clipping’, in others different forms have been distinguished based on the modification action and where it occurs on the leaf or leafy stem (Table 2). While we use four categories, we recognise that many examples may not fall neatly into one distinct category, and these gesture forms may instead represent a gradient with varying levels of visual and audible information. Moreover, as we did not have access to the full dataset (including videos) from previous studies, it is possible that, in practice, there is further variation in form represented in some communities. Variation in these gestures may occur on additional levels, like the body parts used or the number of leaves modified. These considerations are beyond the scope of our current study, but are an ongoing area of research^33,34^.

**Table 2:** Descriptions of four forms of leaf-modifying gestures used in this paper. Video examples of gesture forms can be found in ESM Videos 1–4.

*Form*	Leaf-clip	Leaf-tear	Leaf-pull	Leaf-drop
*Action*				
*Shape of leaf remains*				
Definition*	Rips apart one or more leaves from the midrib and lamina using mouth while holding the leaves in hand. Leaves typically not attached to twig or branch	Tears leaves, one by one, on the midrib, close to the petiole, from a twig with mouth. Typically, while holding twig with hand	Pulls leaves, one by one, from a twig with mouth. Leaves separate from twig at the petiole. Typically, holding twig with hand	Pulls leaves from twig at the petiole or pulls leafy stem from tree and purposely drops or gently throws quietly while sitting in tree

*Emphasis*^+^	Modification	Detachment	Detachment	Release
*Sound*	Distinctive sound	Some tearing sounds	Little to no sound	Little to no sound
Reference	Nishida^24^	Kalan and Boesch^25^	Bessa et al.^18^	Graham et al.^26^

*Labelled diagram of leaf/ leafy stem parts used in descriptions of leaf-modifying gestures.

^+^Emphasis: here we highlight which part of the gestural action includes the most salient leaf-modification (e.g., modification, through ripping, of the leaves during leaf-clipping).

For variation in form to be considered cultural, communities do not need to choose one form and stick to it exclusively. In contrast, groups in which there is clear evidence that individuals can express different forms but *prefer* to use one of them may provide stronger evidence of socially-mediated use. Recent studies^7,35^ suggest chimpanzees show cultural variation in the frequency with which behavioural forms are used. For example, neighbouring communities (with frequent female migration) in the Taï National Park, Ivory Coast, exhibited different *preferences* for hammer tools used for nut cracking^7,35^. While both were observed using stone and wooden hammers, they used these tool types at different frequencies throughout the nut-cracking season^7^. Nuanced variation in the specific form of tool-use, tool manufacturing sequences, or even tool-use posture also exists between communities^36^ suggesting that while the overall behavioural function may be the same, variation in form may also arise as a result of social learning.

Across the *Pan* genus many communities exhibit more than one form of leaf-modifying gesture (Table 1). Using surveys filled out by staff and researchers from different field sites, previous reports described the presence or absence of specific forms of leaf-modifying gestures and, where possible, considered whether these gestures were used habitually^2^. Comparing more detailed preferences for different gesture forms between communities was often challenging as survey methods could not directly account for variation in study methods or levels of habituation between sites^2^. Nonetheless, these initial surveys were essential in identifying the general distribution of leaf-modifying gesture forms across chimpanzee communities.

Leaf-modifying gestures have been described in several behavioural contexts including sexual solicitation^20,22,24,29^, preceding pant-hooting and drumming^18,19,22,25^, requesting food from human researchers in provisioned sites^24^, and while being followed by researchers during habituation^21^; and may be observed in several of these contexts within one community^19,22,24^. Variation in reported use (for example as habitual vs rare) across communities may also be due to variation in the contexts in which they are seen, with some studies focusing on specific contexts—such as buttress drumming^18^. Furthermore, contexts in which leaf-modifying gestures are used in addition to other signals, and those involving a reaction to human observers are naturally more conspicuous to observers, increasing the likelihood of these gestures being recorded in these contexts, rather than those in which leaf-modifying gestures are used in isolation, or where the signaller is moving (e.g., travelling).

Unlike vocal signals, which can be recorded even when the signaller is not visible to observers, gestures, and other visual signals, cannot typically be recorded when the signaller is not fully visible. Observations of visual signals may be more difficult in certain contexts, in dense habitats, or when following unhabituated individuals. However, new tools in primate archaeology allow us to infer a behaviour took place without directly observing them. When chimpanzees perform behaviour that involve objects from their environment (e.g., tool-use or leaf-modifying gestures), material evidence remains after the chimpanzees move to another location, and further investigation of these sites may reveal important details^37,38^. Until now, these techniques have been used to identify the presence and expression of tool-use^38^, buttress drumming^18^, and nesting behaviour^39^. While in most of these cases the observers arrived days or weeks after the chimpanzees had left the site, primate archaeological techniques may also be useful when applied in a shorter time-frame, for example, providing accurate descriptions of the ways in which leaves were modified during gesture production. The four forms of leaf-modifying gestures we describe are conspicuous but very brief, and while their occurrence is easy to detect, the details of the behaviour can be difficult to record on video in a dense forest habitat. However, they leave quite different material remains (Table 2; ESM Figure S1), which we can investigate to confirm gesture occurrence and obtain details about the gesture form.

Here we present the first systematic analysis of leaf-modifying gestures across two neighbouring communities of East African chimpanzees (*Pan troglodytes schweinfurthii*), living in the Budongo Forest Reserve, Uganda. Leaf-modifying gestures (the leaf-clipping form) have already been identified in one of these communities (Sonso) as a gesture used in sexual solicitation, play, and travel^2,29^. We build on new methods, looking beyond presence or absence of specific forms in one community (or across distant communities), to investigate the frequency with which different leaf-modifying gesture forms are expressed in different behavioural contexts, and systematically compare these observations across two neighbouring communities who share a continuous habitat and experience gene flow through regular migration. We implement methods used in primate archaeology to identify the form of leaf-modifying gestures where they could not be reliably determined through direct observation. These new methods and our long study period enable us to systematically quantify inter-community variation in a non-vocal signal in the wild.

## Methods

### Subjects

Waibira and Sonso are wild, habituated communities of East African chimpanzees (*Pan troglodytes schweinfurthii*) living in neighbouring home ranges in the Budongo Forest Reserve in the Western Rift Valley, Uganda (1° 35′ and 1° 55′ N and 31° 08′ and 31° 42′ E). We consider the specific biases in our sample using the STRANGE framework^40^. The Sonso community (~ 70 individuals) is a forest edge community and have been followed by researchers since 1992^41^, while the Waibira community (~ 120 individuals) is a forest bound community, surrounded by other communities, and have been followed by researchers since 2011^42^. While Sonso represent a typically sized community, Waibira is a relatively large community^43^. Furthermore, male mating competition in Waibira is likely considerably higher than in Sonso as the Waibira community have an almost equal male:female adult sex ratio but Sonso have a (more typical) female biased sex ratio with around double the number of adult females to adult males^44^. Both communities are followed daily by a team of trained field assistants who conduct daily focal follows on all independent individuals (those regularly observed in parties without their mother) within the community.

### Data collection

Ad libitum observations of leaf-modifying gestures were recorded by G.B., B.F., A.So., and C.H. between 2007 and 2022 in the Sonso community and between 2011 and 2022 in the Waibira community. Gestural data collection occurred during 69 months in Waibira, and 83 months in Sonso. When possible, observations of leaf-modifying gestures were recorded using a handheld camcorder (Sony Handycam DCR-HC-55; Panasonic VH770; Panasonic HC-VXF1). Otherwise, observations were recorded as an audio description using the same camcorders or with a portable audio recording device.

### Forms of leaf-modifying gestures

Object use is common within the gestural repertoire of chimpanzees, and leaves or leafy stems and branches are often used within gestural communication^29^. We suggest that the leaf-modifying gestures described in this study belong to a distinct category of gestures in which the modification of leaves (through ripping, tearing, pulling, detaching, and/or dropping) is particularly salient within gesture production. When objects are used in other gestures (e.g., object shake, object move, etc.) any resulting modification of leaves is incidental, occurring in only some cases, and not a necessary part of the gesture action. Leaf-modification may also occur outside of the gestural context, for example in grooming behaviour; however, here we focus on variation within chimpanzee gestural communication.

Great ape gestural repertoires are typically constructed by defining gesture ‘types’^26,29,45^. However, there is variation in the level of detail at which a gesture ‘type’ is defined both across and within reported repertoires^46^, such that some are split by context (e.g., present groom and present sexual^29^) or body part (e.g., head shake and arm shake^31^). Furthermore, these are often defined a priori with limited consideration of whether these represent salient, discrete units to the apes employing them (c.f.^33^). In contrast, there has been relatively little description of the degree of morphological variation that could occur within a gesture type (i.e., different gesture expressions or forms). Here we distinguish different leaf-modifying gestures as different *forms* within a broad gesture category. We remain agnostic as to whether these four forms are variants within the same gesture type or represent four different types within a larger category. There may also be additional variation in the form of leaf-modifying gestures and these forms may exist on a graded scale rather than as distinct categories. Thus, our description of form represents a starting point for investigating whether there is group-wise variation within and between neighbouring chimpanzee groups from the same population in the production of these gestures within the same extended habitat.

Building on previous descriptions of the behaviour in other communities (Table 2), we assigned each leaf-modifying gesture observation to one of four possible forms: leaf-clip, leaf-tear, leaf-pull, or leaf-drop. We did not differentiate between the use of hands or mouth in our study because we only recorded one example of an individual using their hands and not mouth to modify leaves. Furthermore, we did not consider the number of repetitive tears/rips or number of leaves used as this was not consistently recorded and was often difficult to determine through videos alone. The form of leaf-modifying gestures was determined in two ways: direct observation and archaeology.

Borrowing from archaeology methods^37–39^, we could determine the form of leaf-modifying gestures after the behaviour occurred by inspecting the shape of the leaves and leaf remains (Table 2; ESM Figure S1). In these cases, while we usually observed or heard the chimpanzee using a leaf-modifying gesture or other gestures, we could not at the time determine the form through direct observation or capture it on video. Instead, once the chimpanzees had moved away, we inspected the location where the gesture took place. If we saw a chimpanzee buttress drumming, we also traced their movement path toward the buttress, searching for remains of fresh leaves, which would indicate a leaf-modifying gesture had been used. By inspecting the shape of the leaves, where/if they were torn, and the remains of the leaves on the vegetation from which they were removed, we could determine the form of leaf-modifying gesture used. For example, where we saw that full leaves were removed from a sapling without tearing, we could infer that the leaf-pull gesture was used. However, if ripped close to the petiole and small parts of the leaves were still attached to the sapling at the petiole, we could infer that the leaf-tear gesture was used. The leaf remains of leaf-drop and leaf-pull were expected to be very similar (i.e., leaves are not torn), and as a result we needed additional information to discriminate these forms, for example: signaller location. Leaf-drop is defined as being produced while the signaller is in a tree^28,29^, thus remains that matched these gesture forms produced where the signaller was on the ground were classified as leaf-pull. Where the signaller was in a tree, direct observation of the gesture production was needed to discriminate leaf-pull from leaf-drop. Where this was not available, we noted the form as “unknown” and excluded it from the data.

The availability of different tools or tool materials within a given habitat may influence the objects used by animals within those environment (e.g., for tool use^47^). The species, size, shape, and freshness of leaves may impact the form of leaf-modifying gestures, if some forms are easier to perform with specific leaves. Previous studies typically compared the use of leaf-modifying gestures between geographically distant communities^2^ (but see Bessa et al^18^), where ecological differences in—for example, tree species—between groups may have contributed to observed differences in the use or expression of leaf-modifying gestures. While we were not able to record the species or size of leaves used in leaf-modifying gestures in this study, the Sonso and Waibira communities live within the same continuous forest habitat with partially overlapping home ranges, and likely had very similar access to tree and shrub species. All cases included in this study involved the use of fresh leaves—plucked directly from the twig during gesturing.

Chimpanzees have been speculated to manipulate the audibility of leaf-modifying gestures^20^. Leaf-clipping is the most audible form as the leaves are repeatedly ripped making a distinctive and sharp sound^24^; leaf-tear is less audible than leaf-clip as each leaf is only ripped once, making some sound, but often the leaf is ripped between the petiole and midrib (Table 2) making less noise as compared to leaf-clip; leaf-pull creates almost no noise as the leaves are plucked directly from the petiole (Table 2) with no ripping; leaf-drop is the most quiet as the leaves or leafy stems are plucked from the tree and dropped silently with no ripping and often including fewer repetitions compared to leaf-pull—though some sound may be produced incidentally as the dropped leaf or leaves disturb vegetation on their descent^26,27^. Finally, the level of audibility may occur on a spectrum and change within gesture forms. To test the hypothesis that chimpanzees manipulate the audibility of leaf-modifying gestures, knowledge of the audience composition, distance to recipient, and precise acoustic recordings are required. As we could not record this information, we are unable to address this question in the current study.

### Behavioural context

We noted the context in which the leaf-modifying gestures occurred (from the perspective of the chimpanzee producing it). We included the following contexts as described in previous reports of leaf-modifying gestures: (1) sexual contexts, including solicitations and consortship^24,48^, (2) preceding pant-hoot and/or drumming^18,19,22^, (3) play^21^, (4) displacement response to human observer^23^, (5) other (e.g., hunting, travel). As chimpanzees in Budongo have never been provisioned we did not consider food requests from humans^24^ as a potential context in these communities. Behavioural contexts were determined by considering the behaviour of the signaller before or after exhibiting leaf-modifying gestures (e.g., copulation, play, or pant-hoot/drum) and by the presence of specific other individuals (e.g., females in oestrus).

### Individual age

Chimpanzees were classified into age categories following Reynolds^41^ with adults including all individuals older than 16 years, subadults between ten and 16 years, juveniles between five and ten years, and infants younger than five years. However, as clear recordings of leaf-modifying gestures were relatively rare in these communities, we subsequently lumped the age categories into mature (> 10 years) and immature (< 10 years).

### Analysis

We were unable to formally compare the use of leaf-modifying gestures across contexts as ad libitum observations were likely biased towards contexts in which gestures were more conspicuous. For example, in sexual contexts, regular repeated gestures as part of long gestural sequences occur throughout an interaction, whereas in drumming episodes a single brief gesture at the onset of drumming may be hard to detect. We therefore report a descriptive account of differences and similarities in the contexts in which leaf-modifying gestures were used and the age and sex categories of individuals who were observed using them.

We compared the frequencies in forms of leaf-modifying gestures observed between Waibira and Sonso communities using Chi-square tests^49^ in R version 4.04^50^. For this test we categorised each leaf-modifying gesture event into one of two forms: leaf-clip and leaf-pull/tear to increase our sample size for each category. We decided to group the two forms leaf-pull and leaf-tear as individuals were observed using both forms of leaf modification within one gesture event (continuous repetition of tearing and/or pulling without pause) on several occasions, making it difficult to accurately discriminate such events into one of the two categories. Furthermore, while one possible interpretation is that there are two forms of the gesture that are used in combination, a more conservative interpretation is that the combination of pulling and tearing within the same gesture event may suggest that leaf-pull and leaf-tear do not represent two distinct gesture forms for the Waibira chimpanzees. We excluded one observation of leaf-drop from this analysis as it was only observed on a single occasion and may therefore represent an outlier or accidental production.

### Ethical statement

Ethical approval for this project was obtained from the University of St Andrews Animal Welfare and Ethics Committee under approval code PS15842. Data collection protocols were observational and followed the International Primate Society code of Best Practice for Field Primatology^51^.

## Results

We recorded 30 cases of leaf-modifying gestures in the Waibira community and 41 cases in Sonso (ESM Table S1) in which we could discriminate the context and/or type of leaf modification used. Fourteen individuals (12 males, two females) in Waibira and 21 individuals in Sonso (12 males, nine females) contributed to these observations.

### Individual age

**Figure 1:**
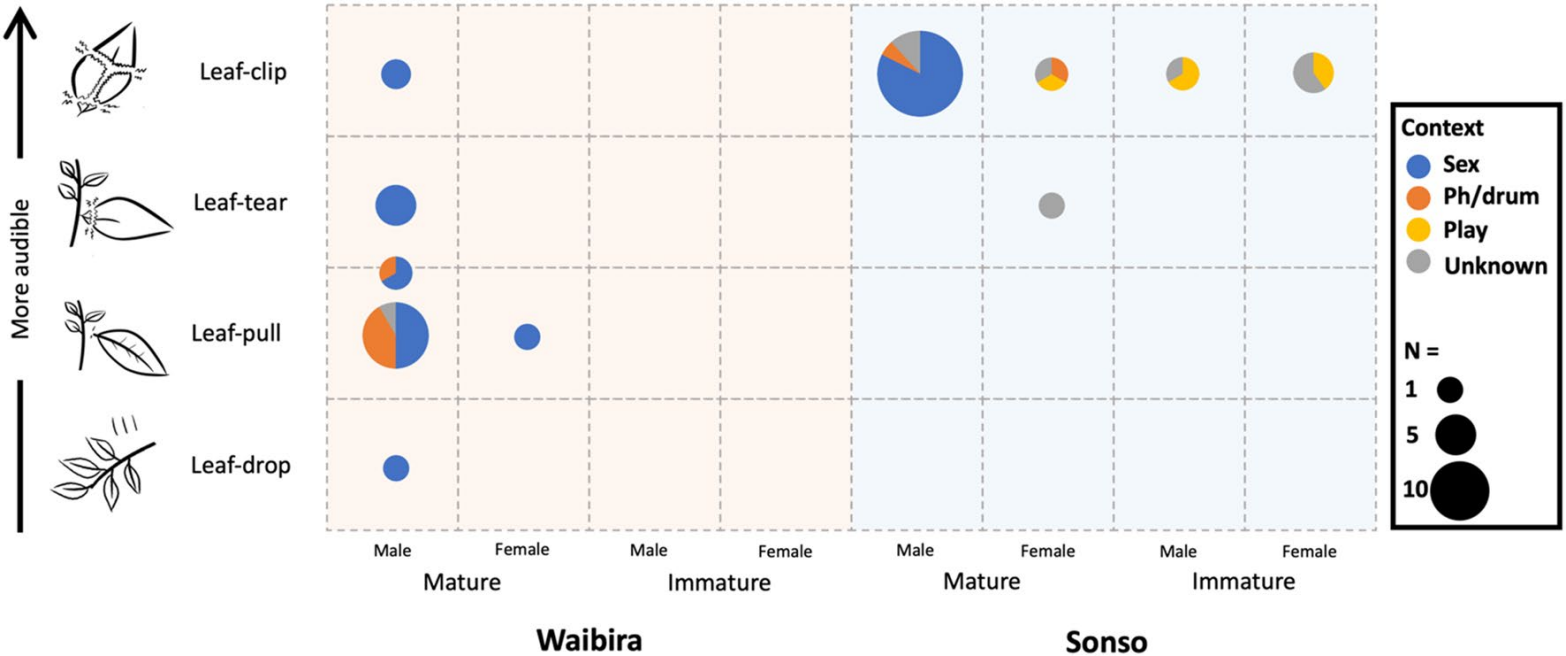
Number of observations of each leaf-modifying gesture form in each community by age/sex category. Intermediate forms (i.e., cases in which multiple forms are used within the same event) are presented in-between columns of two forms. (e.g., leaf-pull/leaf-tear). Illustrations show what the leaves look like after they have been modified. Moving from the bottom to the top these different forms become more audible. Pie chart colours represent the proportion of cases observed in each behavioural context (Sex, Pant-hoot/drumming, Play, or Unknown).

In both communities (Fig. 1) most leaf-modifying gestures were performed by mature males (Waibira N = 28, 93%; Sonso N = 23, 56%), although mature females also used these gestures at both sites (Waibira N = 2, 7%; Sonso N = 7, 17%). Immature individuals were only observed using leaf-modifying gestures in Sonso (females N = 8, 19%; males N = 3, 7%).

### Context

Most leaf-modifying gestures were observed in the sexual context in both communities (Waibira = 67%; Sonso = 49%; Table 3). Leaf-modifying gestures were also observed preceding pant-hoots/drumming in both communities (Waibira = 23%; Sonso = 7%) but were more common in Waibira (Table 3). Only in Sonso, were leaf-modifying gestures also observed in play solicitation (22%; Table 3). In Sonso, one adult female (2%) appeared to use a leaf-modifying gestures as a travel initiation before drumming (included in context = “other” and “Pant-hoot/drum”; Table 3). We were not able to identify a clear behavioural context in three cases for Waibira, and nine cases for Sonso (Table 3).

**Table 3:** Number of observations of leaf-modifying gestures recorded in each context in the Waibira and Sonso chimpanzee communities.

	(1) Sex	(2) Pant hoot/drum	(3) Play	(4) Human observer	(5) Other	Unknown
Waibira	20 (67%)	7 (23%)	0	0	0	3 (10%)
Sonso	20 (49%)	3 (7%)	9 (22%)	0	1 (2%)	9 (22%)

Numbers correspond to context descriptions detailed in the methods section and headers are short hands for these descriptions.

### Form of leaf-modifying gestures

Of the total 71 cases, we were able to accurately determine the form of leaf-modifying gestures in 53 (75%) observations (Waibira N = 24, 80%; Sonso N = 29, 71%). When comparing the leaf clip, and leaf tear/pull, Sonso and Waibira exhibited apparent preferences for different gesture forms (N = 52, X^2^ = 37.043, *df* = 1, *p* value < 0.001; Fig. 1). Sonso predominantly used the leaf-clip form (N = 28, 97%), while Waibira predominantly used the leaf-tear/pull form (N = 21, 91%). Across all but one observation, individuals in both communities used their mouth to perform leaf-modifying gestures. In Sonso, one immature male was observed leaf-clipping with his hands. In Waibira, individuals sometimes combined leaf-tearing and leaf-pulling within the same gesture event. All cases reported here involved the use of fresh leaves, however, one male in the Sonso community (FK) was previously observed to use dry leaves on one occasion (CH pers. obs.) when using the leaf-clipping form before pant-hooting/ drumming. Both young and mature leaves from a range of tree species were used within and across gesture forms. All these cases involved repetitive ripping or removal of leaves from the petiole on a single twig, but some leaves were audibly removed by tearing across the petiole, and others were silently removed by pulling off the stem at the petiole.

## Discussion

Variation in communication between social groups or populations (dialects) has been reported predominantly in non-human vocal signals^1,10,12^, with few studies exploring non-vocal dialects outside human communication (e.g., honey bee waggle dance^15^). Leaf-modifying gestures have previously been suggested as a possible cultural behaviour in the *Pan* genus^2,18,19,23,25^. However, the study of potential cultural differences is hampered where the nuances of behavioural expression remain unclear. We investigated this behaviour with a systematic analysis of leaf-modifying gestures—a non-vocal signal—in two neighbouring communities of East African chimpanzees. We found that while the contexts in which leaf-modifying gestures are used were similar in both communities, the preferred form of leaf-modifying gesture expression varied. The Sonso community show a strong preference for the leaf-clip form, while the Waibira community show a bias towards the leaf-pull and leaf-tear forms.

Considering all four forms of leaf-modifying gestures, it appears that the Waibira community show a greater variety of forms (i.e., they exhibit all four forms). However, the combination of leaf-pull and leaf-tear within multiple gesture events suggests that a categorical distinction between these two forms may not be meaningful to the chimpanzees in this community. The difference between leaf-pull and leaf-tear may represent a different degree of variation than the difference between, for example, leaf-clip and leaf-pull. At present, we continue to refer to these variants as different ‘forms’, but note the caveat that they may not represent variation on the same scale. Furthermore, while it is a well described gesture form in bonobos, the rare (n = 1) observation of leaf-drop may represent an anomaly or accidental production in Budongo. With this context in mind, each community appears to habitually use two forms of leaf-modifying gestures and show opposite preferences for one over the other.

While establishing absence is challenging, it is noteworthy that despite long-term research with a focus on gestural communication ongoing for > 15 years, there have been no observations of the leaf-pull or leaf-drop forms, and only very rare observations of the leaf-tear form in the Sonso community, whereas all four forms have already been observed in the more recently habituated Waibira community. Thus, the apparent differences in the use of gesture forms between the groups appear likely to be based in the chimpanzees’ behaviour. Furthermore, preliminary evidence suggests different developmental trajectories in the use of leaf-modifying gestures: with individuals in Sonso, but not in Waibira, regularly using these gestures before reaching maturity. Our results support the argument that some aspects of leaf-modifying gesture use vary between neighbouring chimpanzee communities and may be socially derived.

In both communities, leaf-modifying gestures were used across a range of similar behavioural contexts. Given the diversity of contexts in which leaf-modifying gestures are used, it has been suggested that these gestures may be linked to a state of ‘frustration’ present across contexts and/or used as displacement behaviours^21,24,25^. While the gestures in our study were intentionally produced, they may also be used in contexts in which frustration is more likely. For example, producing loud, long-distance, broadcast signals like pant-hoots and drumming is more risky when social tension is high in the community (e.g., in the South Group of chimpanzees at Taï^25^). One could test whether gestures used during more frustrating contexts are produced more rapidly, with greater emphasis, or as part of longer bouts (as in other mammals^52–54^).

While Sonso and Waibira occupy neighbouring (and partly overlapping) territories in the same forest and experience regular migration, there are some differences between them that may be salient for gesture production. The Waibira community is approximately double the size of Sonso with a considerably higher male to female adult sex ratio (Waibira = 1:1, Sonso = 1:2), which likely increases mating competition between males^44^. Higher mating competition between Waibira males may provide an explanation for the apparent delay in the onset of these gestures’ use, particularly in sexual solicitation, as immature individuals are not yet able to compete for access to sexually receptive females^55,56^. An alternative explanation may be that the shorter observation time and slightly lower habituation of some female and immature individuals in Waibira could have resulted in fewer observations of rare behaviour. Nonetheless, even if immature individuals in Waibira do use leaf-modifying gestures they are unlikely to use them regularly; the community has been followed daily for over a decade, and even the immature offspring of extremely well habituated Sonso born females who emigrated to Waibira (N = 3) have not yet been observed using these gestures. Finally, while the use of quieter gesture forms in Waibira (e.g., leaf-pull), as compared to Sonso (leaf-clip), may be an intentional strategy to avoid detection by eavesdroppers or competitors who may interrupt the behaviour^20,57–59^, as observed in other animals^60^ (e.g., lions^61^); leaf-modifying gestures congruent with community preferences were also used outside of sexual contexts in both Waibira and Sonso, suggesting that different gestural preferences in these communities are unlikely to be fully explained by variation in their social demographics. Future studies could consider audience effects on gestural production in these contexts to explicitly test this hypothesis.

The preferences for different leaf-modifying gesture forms in Sonso and Waibira, together with the common use of these gestures in the same context towards a similar goal (sexual solicitation), suggests that the observed variations in gesture form could be better described as a variation in gesture expression rather than different gesture types, and that these preferences could be shaped by local culture. The repertoire of gesture types available to great apes has been suggested to be genetically endowed and shared within, and largely across, great ape species^32^; however, the degree to which gesture production is shaped by an apes’ social environment is only starting to be described. For example, there is evidence for preferential use of gesture types from within the available repertoire (e.g., *shake back, turn bipedal,* and *rear up* in travel initiation^62^). Beyond gesture ‘types’, an area that remains to be explored is more nuanced variation of gestural expression and morphology in non-human animals—in humans, the kinetics (the body parts, plane of gesture space, and gesturing tempo), geography of conversation (how participants gesture to one another), and how gestures relate to the language they accompany, all vary between cultures^63,64^. A similar capacity for cultural conformity is already described in chimpanzee social customs, like hand clasping^65^, and in chimpanzee tool-use. In the North, South, and East groups of Taï chimpanzees, immigrant females adapt their nut-cracking to the use of different hammer materials (e.g., wooden or stone hammers) within a tool-use context, even where the newly adopted form may be less efficient^7^.

Variation in behaviour between animal groups may also be driven by local ecology (e.g., cricket frogs mating call^66^, honey bee waggle dance^15^). Thus, variation in the form of gestures involving objects could be a function of the objects available in the environment^47^. While the Waibira and Sonso communities live in the same continuous forest, micro-habitats within each community home range could make leaves of certain species more or less available in a given community, thereby shaping the use of different leaf-modifying gesture forms. However, fresh leaves were highly preferred over dry leaves in both communities (except for one anecdotal example of dry leaves use), and selection of leaves ranged between young leaves of saplings and mature leaves in trees within and across gesture forms. Furthermore, in Waibira the diverse gesture forms were used within the same community range (and at times in the same gesturing event). Taken together, these observations suggest that the variation in form observed between communities is unlikely to be driven mainly by differences in ecology, even if we cannot entirely exclude the possibility that a more detailed analysis may reveal small differences in leaf species, shape, or size between gesture forms.

Socially derived variation in the expression of the same signal between groups (‘dialects’) is described across several bird and cetacean species^1,8,9,67^, and has been reported in chimpanzee vocalisations, where group differences were shown in the expression of pant-hoot vocalizations^13,16^. Recent work suggesting promising avenues for the exploration of cultural variation in chimpanzee communication propose that the apparent context-specific use of leaf-clip gestures in some communities may reflect gesture ‘dialects’ too^23^.

Dialects may be a form of cultural variation when they arise through social conformity to the communicative habits of other individuals in the same social group, but which may also interact with local variation in genetic and ecological factors all of which contribute to inter-group variation^13,15^. It is still unclear when inter-group signal variation in non-human communication constitutes different dialects as opposed to other forms of variation; for example, more nuanced variation akin to an accent, or more profound lexical and structural variation, akin to a distinct language. Human languages and birdsongs of populations that are geographically closer also tend to exhibit more similar dialects^11,67^; and both may be partially shaped by local ecology^68,69^. We do find some (limited) overlap in the expression of leaf-modifying gestures between Waibira and Sonso, with both communities exhibiting the leaf-clip and leaf-tear forms of the behaviour, as well as overlap in the contexts in which leaf-modifying gestures are used in both communities. It is still unclear to what extent overlap between Waibira and Sonso leaf-clipping is a product of their shared habitat and/or cultural transmission between groups.

The Sonso and Waibira communities live in the same habitat and experience gene flow, including through female migration of habituated individuals from Sonso to Waibira. Community preferences for different gesture forms of leaf-modifying gestures appear stable across 10–15 years of data collection, and do not appear to be explained by variation in observation effort. While we did not observe females who emigrated between the communities using leaf-modifying gestures before and after migration, at least two females (BAH and NOR; ESM Table S1) were solicited for sex in both communities and were successfully solicited with a different form—consistent with the community preference. Their ability to understand both forms may indicate some flexibility in gesture comprehension in mature individuals—perhaps helped by the similarity in gesture forms, and the (occasional) use of non-preferred forms in both communities. This pattern resembles that observed in common marmosets, who adopted the vocal dialect of a new group after translocation^12^.

Our results add to a growing literature on culture and behavioural flexibility in non-human animal communication outside of the vocal domain. While the term ‘leaf-clipping’ has previously been used to refer to a broad category of gestures used across a variety of contexts and different chimpanzee communities, it represents just one of several forms of leaf-modifying gestures that together offer substantial scope for variation to be expressed. Our ability to describe patterns of variation in gesture use is dependent on accurate and nuanced descriptions of gesture production that go beyond presence and absence of gesture ‘types’. Some variation, like the development of gesture use, between chimpanzee communities may be shaped by differences in the social environment, for example in male mating competition. However, conformity to local customs likely shape gesture production and comprehension, including in adult migrant females, suggesting that there is scope for flexibility and socially-mediated learning of local patterns of gesture use that may represent cultural ‘dialects’. Future research should focus on disentangling the effects of culture and selective pressures on non-vocal expression and further explore the flexibility of non-vocal signals as a means to describe cultural variation across species.

## Supplementary Information

Supplementary Information 1.

Supplementary Video 1.

Supplementary Video 2.

Supplementary Video 3.

Supplementary Video 4.

Supplementary Information 2.

## Data Availability

Data are available in ESM Table S1.
