# Supplementary figures and images for: Dialects in leaf-clipping and other leaf-modifying gestures between neighbouring communities of East African chimpanzees
Source: Sci Rep. 2023 Jan 5;13:147. doi: 10.1038/s41598-022-25814-x (PMC9814361; doi:10.1038/s41598-022-25814-x)

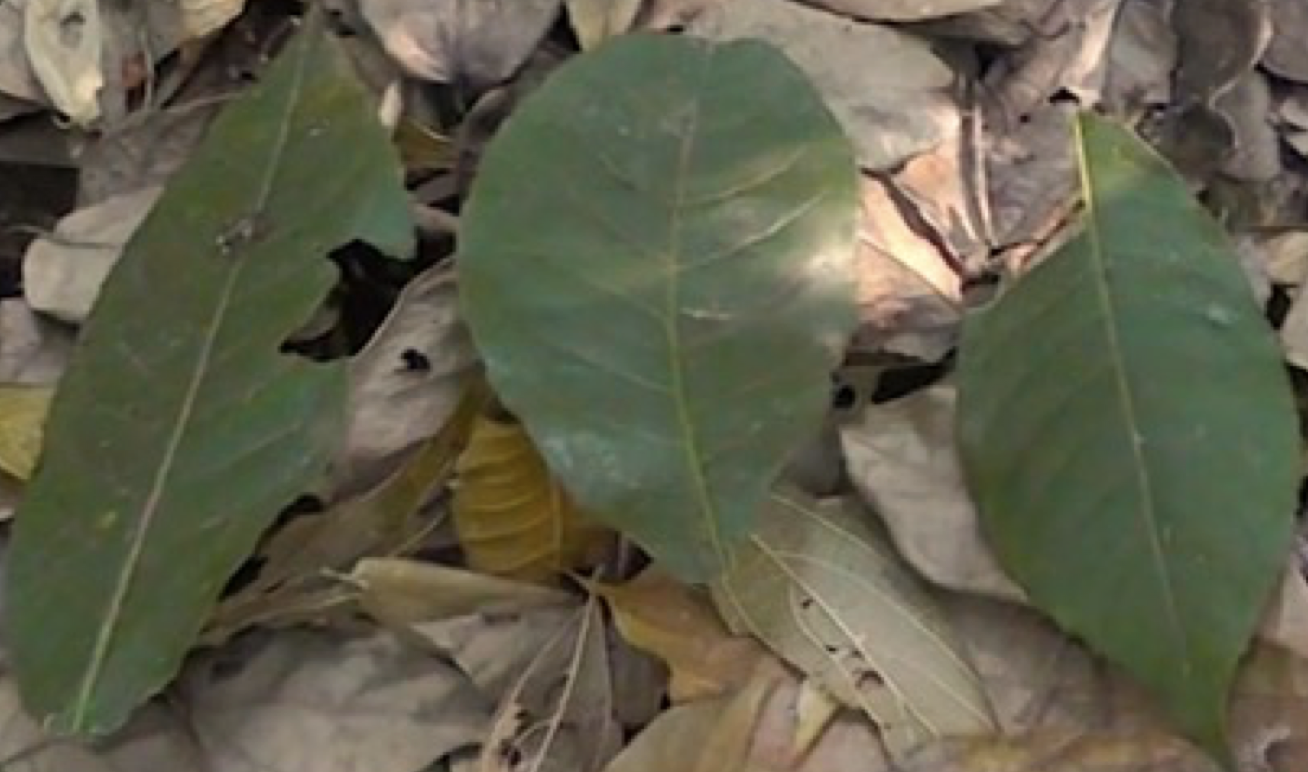

Supplement: Supplementary file 1 — Supplementary Information 1. [file 41598_2022_25814_MOESM1_ESM.png]
